# Elucidation of the effects of a high fat diet on trace elements in rabbit tissues using atomic absorption spectroscopy

**DOI:** 10.1186/1476-511X-9-2

**Published:** 2010-01-12

**Authors:** Mohamed Anwar K Abdelhalim, Hisham A Alhadlaq, Sherif Abdelmottaleb Moussa

**Affiliations:** 1 Department of Physics and Astronomy, College of Science, King Saud University, Saudi Arabia; 2 Department of Science, King Khalid Military College, Saudi Arabia

## Abstract

**Background:**

The mechanism of atherogenesis is not yet fully understood despite intense study in this area. The effects of high fat diet (HFD) on the changes of trace elements [iron (Fe), copper (Cu) and zinc (Zn)] in several tissues of rabbits have not been documented before. Thus, the aim of this study was to elucidate the changes in trace elements in several tissues of rabbits fed on HFD for a period of feeding of 10 weeks.

**Results:**

The HFD group was fed a NOR rabbit chow supplemented with 1.0% cholesterol plus 1.0% olive oil. Fe, Cu and Zn concentrations were measured in four types of tissue from control and HFD rabbits using atomic absorption spectroscopy (AAS). Comparing HFD rabbits to control rabbits, we found that the highest percentage change of increase of Fe was 95% in lung tissue, while the lowest percentage change of increase of Fe was 7% in kidney tissue; the highest percentage change of decrease of Cu was 16% in aortic tissue, while the lowest percentage change of decrease of Cu was 6% in kidney tissue; and the highest percentage change of decrease of Zn was 71% in kidney tissue, while the lowest percentage change of decrease of Zn was 8% in lung tissue.

**Conclusions:**

These results suggest that Fe plays a major role in atherogenesis; it may accelerate the process of atherosclerosis probably through the production of free radicals, deposition and absorption of intracellular and extracellular lipids in the intima, connective tissue formation, smooth muscle proliferation, lower matrix degradation capacity and increased plaque stability. Furthermore, inducing anemia in HFD rabbits may delay or inhibit the progression of atherosclerosis. Cu plays a minor role in atherogenesis and Cu supplements may inhibit the progression of atherogenesis, perhaps by reducing the migration of smooth muscle cells from the media to the intima. Zn plays a major role in atherogenesis and that it may act as an endogenous protective factor against atherosclerosis perhaps by reducing lesion Fe content, intracellular and extracellular lipids in the intima, connective tissue formation, and smooth muscle proliferation. These results suggest that it may be possible to use the measurement of changes in trace elements in different tissues of rabbits as an important risk factor during the progression of atherosclerosis.

## Background

Atherosclerosis is a disease of large- and medium-sized arteries, and is characterized by endothelial dysfunction (malfunction of the cells lining the inside of the artery wall), vascular inflammation, migration of smooth muscle cells to the inner lining of the artery (intima) and the build-up of lipids, cholesterol and cellular debris within the intima of the vessel wall. The biological mechanisms by which low density lipoprotein (LDL) promote formation of atherosclerotic plaques are still poorly understood. Oxidation of LDL has been found to increase its uptake in macrophages and lead to formation of macrophage foam cells. Other studies have indicated that oxidized LDL may induce vascular inflammation and even give rise to autoimmune reactions in the vascular wall. These activated macrophages produce numerous factors that are injurious to the endothelium, leading to plaque formation. In later stages, calcification in the damaged region leads to hardening of the artery wall, coupled with acute and chronic arterial obstruction [1-3].

Fe may participate in diverse pathological processes by catalyzing the formation of reactive oxygen free radicals. The oxidation of LDL and lipid is believed to be one of the crucial events leading to plaque formation in vasculature. It has been hypothesized that iron-mediated oxidation is involved in this process. Several epidemiological studies have shown that the level of body Fe stores is positively correlated with the incidence of coronary heart disease in human populations. Additional experiments in animals have further revealed that the severity of atherosclerosis can be markedly influenced by Fe overload or deficiency [4,5].

Cu supplements inhibit the progression of atherosclerosis by increasing superoxide dismutase (SOD) expression, thereby reducing the interaction of nitric oxide (NO) with superoxide, and hence potentiating NO-mediated pathways that may protect against atherosclerosis [6].

Zn supplementation decreased the elevated levels of cholesterol oxidation products in the aorta and plasma caused by eating a high-cholesterol diet. Several studies have shown that Zn reduced oxidative damage and the risk of cardiovascular disease. Scientists have suggested that because Zn supplementation both reduced the formation of atheromas and lowered lipid peroxidation it may have antioxidant activity. Since Zn is not redox active, it may not act directly as a scavenging antioxidant but instead may act as an indirect antioxidant by competing with pro-oxidant metals such as Fe and Cu for strategic binding sites [7-9]. The role of a high fat diet (HFD) on trace elements [iron (Fe), copper (Cu) and zinc (Zn)] in different tissues of rabbits has, in general, not been studied. Thus, the aim of this study was to elucidate the effects of a HFD on trace elements (Fe, Cu and Zn) in different tissues of rabbits using atomic absorption spectroscopy (AAS).

## Methods

### Rabbit tissue samples

The atherosclerotic model used in this study was the New Zealand white rabbit (male, 12 weeks old), obtained from the Laboratory Animal Center (College of Pharmacy, King Saud University). Twenty rabbits were individually caged, and divided into control group and HFD group. The control group (n = 8) was fed on 100 g/day of NOR diet (Purina Certified Rabbit Chow # 5321; Research Diet Inc., New Jersey, USA) for a period of 10 weeks. Chemical composition of the laboratory NOR rabbit diet (Purina Certified Rabbit Chow # 5321) is shown in Table 1 and Table 2. The HFD group (CHO; n = 12) was fed on NOR Purina Certified Rabbit Chow # 5321 supplemented with 1.0% cholesterol plus 1.0% olive oil (100 g/day) for the same period of time. The animals were sacrificed by intravenous injection of Hypnorm (0.3 ml/kg) in accordance with the guidelines approved by King Saud University Local Animal Care and Use Committee. To obtain protoplasm representative of the *in vivo *situation and to avoid autolysis changes and bacterial growth, the aortas, hearts, lungs and kidneys were carefully removed in a manner which avoided any damage to the tissues. Each segment was rapidly flushed with deionized water to remove any residual blood. The tissue samples were flash-frozen in liquid nitrogen and stored at -85°C until analysis.

**Table 1:** Chemical composition of laboratory NOR diet (Nutrients; Purina Certified Rabbit Chow # 5321)

Nutrients			
Protein%	16.20	Cholesterol, ppm	0.00

Arginine%	0.84	Fat (acid hydrolysis)%	4.00

Cystine%	0.25	Linoleic Acid%	1.31

Glycine%	0.77	Linolenic Acid%	0.08

Histidine%	0.38	Arachidonic Acid%	0.00

Isoleucine%	0.88	Omega-3 Fatty Acids%	0.08

Leucine%	1.30	Total Saturated Fatty Acids%	0.43

Lysine%	0.78	Total Monounsaturated Fatty Acids%	0.70

Methionine%	0.35	Fiber (Crude)%	14.00

Phenylalanine%	0.80	Neutral Detergent Fiber%	27.40

Tyrosine%	0.50	Acid Detergent Fiber%	17.10

Threonine%	0.64	Nitrogen-Free Extract (by difference)%	50.00

Tryptophan%	0.14	Starch%	21.50

Valine%	0.84	Glucose%	0.34

Serine%	0.85	Fructose%	0.90

Aspartic Acid%	.87	Sucrose%	2.44

Glutamic Acid%	3.33	Lactose%	0.00

Alanine%	0.85	Total Digestible Nutrients%	66.00

Proline%	1.31	Gross Energy, kcal/gm	3.81

Taurine%	<0.01	Physiological Fuel Value, kcal/gm	2.88

Fat (ether extract)%	2.50	Metabolizable Energy, kcal/gm	2.49

Purina Certified Rabbit Chow # 5321; Research Diet Inc., New Jersey, USA).

**Table 2:** Chemical composition of laboratory NOR diet (Minerals and Vitamins; Purina Certified Rabbit Chow # 5321)

Minerals		Vitamins	
Ash%	7.30	Carotene, ppm	28.00

Calcium%	1.10	Vitaimn K, ppm	2.90

Phosphorus%	0.50	Thiamin Hydrochloride, ppm	4.80

Phosphorus (non-phytate)%	0.27	Riboflavin, ppm	5.00

Potassium%	1.20	Niacin, ppm	54.00

Magnesium%	0.25	Pantothenic Acid, ppm	19.00

Sulfur%	0.24	Choline Chloride, ppm	1600.00

Sodium%	0.30	Folic Acid, ppm	8.40

Chlorine%	0.66	Pyridoxine, ppm	4.50

Fluorine, ppm	11.00	Biotin, ppm	0.20

Iron, ppm	340.00	B_12 _mcg/kg	6.60

Zinc, ppm	120.00	Vitamin A, IU/gm	20.00

Manganese, ppm	121.00	Vitamin D, IU/gm	1.10

Copper, ppm	17.00	Vitamin E, IU/gm	44.00

Cobalt, ppm	0.50	Ascorbic Acid, mg/gm	-

Iodine, ppm	1.10	-	-

Chromium, ppm	0.70	-	

Selenium, ppm	0.25	-	-

Purina Certified Rabbit Chow # 5321; Research Diet Inc., New Jersey, USA).

### Digestion of rabbit tissue samples

Various rabbit tissue samples were wet digested with nitric acid and converted into acidic digest solutions for analysis by AAS method. The tissue was freeze dried in order to minimize loss of analytes and to facilitate subsequent sample preparation steps, and then homogenized to a fine powder by ball-milling in plastic containers. Approximately 0.20 to 0.25 g of powdered tissue was weighed into a Teflon reaction vessel and 3 ml of HNO3 were added. The closed reaction vessel was heated in a 130°C oven until digestion was completed. Samples were then diluted to a final volume of 20 ml with quartz distilled water and stored in 1 oz. polyethylene bottles for later analysis by instrumental techniques.

### AAS measurements

AAS determines the presence and concentration of trace elements [iron (Fe), copper (Cu) and zinc (Zn)] in different tissues of rabbits. Fe, Cu and Zn absorbed ultraviolet (UV) light when they were excited by heat. The AAS instrument looks for a particular metal by focusing a beam of UV light at a specific wavelength through a flame and into a detector. The sample of interest was aspirated into the flame. If that metal is present in the sample, it will absorb some of the light, thus reducing its intensity. The instrument measures the change in intensity. A computer data system converted the change in intensity into an absorbance. As concentration goes up, absorbance goes up. A calibration curve was constructed by running standards of various concentrations (10, 15 and 20 PPM) on the AAS and observing the corresponding absorbance. A calibration curve was made and then samples were tested and measured against this curve. AAS measurements were carried out at the Research Center for Girls, King Saud University. Trace elements (Fe, Cu and Zn) were measured using a Specter AA-220 series double-beam digital atomic absorption spectrophotometer. The concentration of trace elements in each tissue sample was calculated by comparing the absorbance produced by the sample with that produced by a series of standards as follows:

### Statistical analysis

The results were expressed as mean ± standard error (SE). To assess the significance of the differences between the control group and HFD group of rabbits, statistical analysis was performed using one-way analysis of variance (ANOVA) for repeated measurements, with significance assessed at 5% confidence level.

## Results

**Figure 1:**
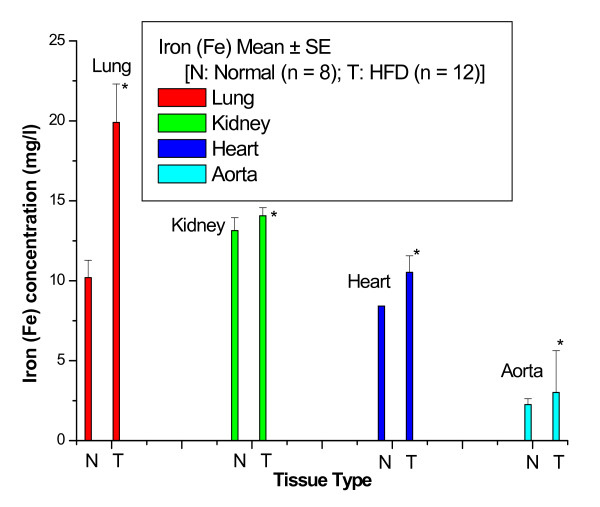
**Fe concentrations in lung, kidney, heart and aortic tissues of control and HFD rabbits**.

Figure 1 shows the Fe concentrations in lung, kidney, heart and aortic tissues of control and HFD rabbits. The Fe concentration was significantly increased with percentage changes of 95% in lung, 7% in kidney, 25% in heart and 33% in aorta of HFD rabbits compared with control rabbits.

**Figure 2:**
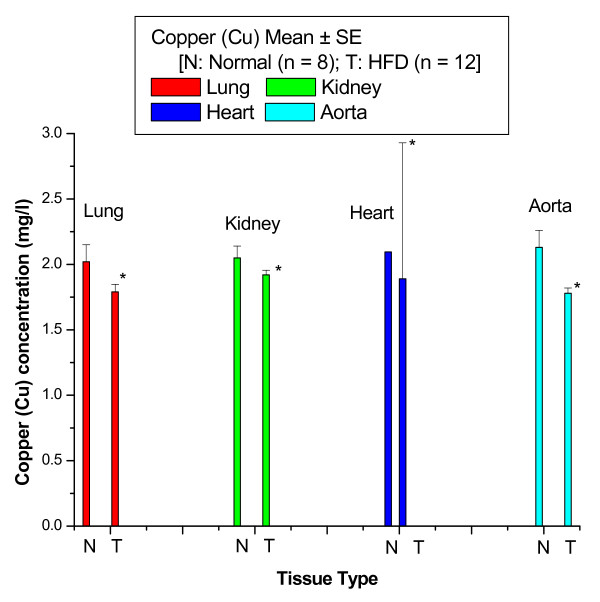
**Cu concentrations in lung, kidney, heart and aortic tissues of control and HFD rabbits**.

Figure 2 shows the Cu concentrations in lung, kidney, heart and aortic tissues of control and HFD rabbits. The Cu concentration was significantly decreased with percentage changes of 11% in lung, 6% in kidney, 9% in heart and 16% in aorta of HFD rabbits compared with control rabbits.

**Figure 3:**
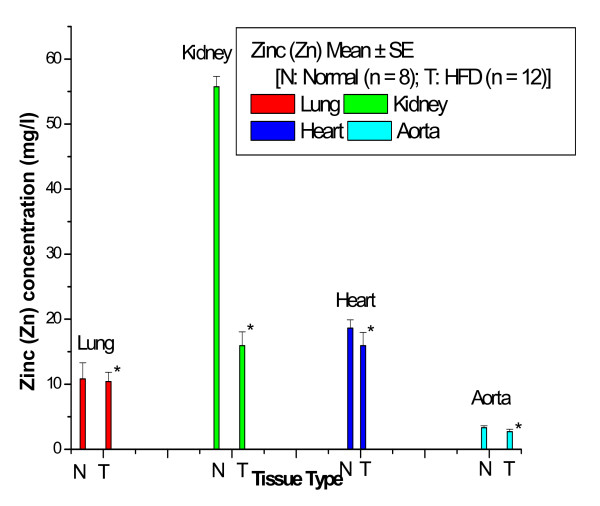
**Zn concentrations in lung, kidney, heart and aortic tissues of control and HFD rabbits**.

Figure 3 shows the Zn concentrations in lung, kidney, heart and aortic tissues of control and HFD rabbits. The Zn concentration was significantly decreased with percentage changes of 8% in lung, 71% in kidney, 14% in heart and 18% in aorta of HFD rabbits compared with control rabbits.

Figures 1 to 3 show that the highest percentage change of increase of Fe was 95% in lung tissue, while the lowest percentage change of increase of Fe was 7% in kidney tissue; the highest percentage change of decrease of Cu was 16% in aortic tissue, while the lowest percentage change of decrease of Cu was 6% in kidney tissue; and the highest percentage change of decrease of Zn was 71% in kidney tissue, while the lowest percentage change of decrease of Zn was 8% in lung tissue.

**Figure 4:**
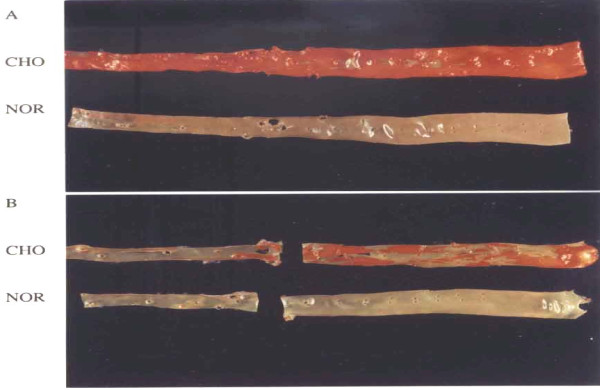
**Photomicrograph of Sudan-stained whole aorta from NOR and CHO**.

Figure 4 shows photomicrograph of Sudan-stained whole aorta from NOR and CHO. To clarify the degree of fatty streaks and fibrous plaques, specimens from the aorta of NOR and HFD (CHO; fed HFD for a feeding period of 10 weeks) were stained with Sudan as shown in Fig. 4 (panel A: thoracic aorta; panel B: abdominal aorta). The aorta of NOR rabbits were completely free of fatty streaks and fibrous plaques, and were characterized by a barely visible intima. On the contrary, all aortic specimens from CHO rabbits exhibited lesions which comprised of fatty streaks and fibrous plaques.

**Figure 5:**
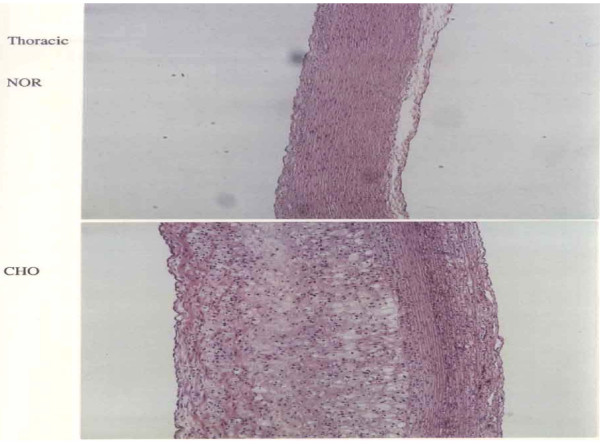
**Photomicrograph of hematoxylin and eosin-stained aorta from a NOR and a CHO**.

Figure 5 shows photomicrograph of hematoxylin and eosin-stained thoracic aorta from a NOR and a CHO. To clarify the degree of atherosclerotic lesions, specimens from the aorta of NOR and CHO were stained with hematoxylin and eosin. The upper panel NOR illustrates normal arterial wall morphology. The lower panel CHO shows marked intimal thickening with focal loss of medial architecture. The intima contains intracellular and extracellular lipids, connective tissue formation, and smooth muscle proliferation.

## Discussion

In this study, HFD rabbits were fed normal rabbit chow supplemented with 1.0% cholesterol plus 1.0% olive oil for a period of 10 weeks. We found that in HFD rabbits, the percentage change of Fe was significantly increased in lung by 95%, kidney by 7% heart by 25% and aorta by 33% compared with control rabbits. These results suggest that Fe plays a major role in atherogenesis, probably through the production of free radicals, and that inducing anemia in HFD rabbits may delay or inhibit the progression of atherosclerosis. This study proposes that percentage increase in Fe in several tissues of rabbit may enhance deposition and absorption of intracellular and extracellular lipids in the intima, promote connective tissue formation, and accelerate smooth muscle proliferation leading to lower matrix degradation capacity and increased plaque stability. To clarify the degree of fatty streaks and fibrous plaques, specimens from the aorta of NOR and CHO were stained with Sudan. The aorta of NOR rabbits were completely free of fatty streaks and fibrous plaques, and were characterized by a barely visible intima. On the contrary, the aortic specimens from CHO rabbits exhibited lesions which comprised of fatty streaks and fibrous plaques. Furthermore, to clarify the degree of atherosclerotic lesions, specimens from the aorta of NOR and CHO were stained with hematoxylin and eosin. the aortic specimens from CHO rabbits illustrates marked intimal thickening with focal loss of medial architecture. The intima contains intracellular and extracellular lipids, connective tissue formation, and smooth muscle proliferation.

Several epidemiological studies have investigated the role of Fe as a potential risk factor in coronary heart disease, although many of this type of study have conflicting conclusions [10]. It has been reported that premenopausal women suffered a lower incidence of coronary heart disease compared with men of the same age because of their lower body Fe storage [11]. Any unregulated Fe has the potential to catalyze and generate hydroxyl radicals from superoxide and hydrogen peroxide via the Fenton reaction. The highly reactive hydroxyl radicals subsequently cause lipid peroxidation, degradation of other macromolecules, leading to cell damage or death [12]. In the study by Lee et al. [13] using apo E-deficient mice, vascular Fe deposition was shown to be closely related to the progression of atherosclerosis and LDL oxidation. Watt et al. [14] have indicated that inducing mild anemia in cholesterol-fed rabbits decreases the progression of atherosclerosis, in conjunction with decreases in lesion Fe content. In another study, rabbits fed with a HFD for 12 weeks, with desferal administration for the final nine weeks, exhibited a significant reduction in average lesion area as compared with 12-week HFD controls [15].

In this study, percentage change of Cu was significantly decreased in lung by 11%, kidney by 6% heart by 9% and aorta by 16% in HFD rabbits compared with control rabbits. These results suggest that Cu may catalyze free radicals through the Fenton reaction, and that it plays a minor role in atherogenesis because its percentage change is lower than the percentage change of Fe in most of the tissues of HFD rabbits. Furthermore, these results suggest that Cu supplements may inhibit the progression of atherogenesis, perhaps by reducing the migration of smooth muscle cells from the media to the intima. Watt et al. [14] have reported that Cu is depleted in the early lesion, at an average level of ~3 ppm compared with ~6 ppm of adjacent artery wall, while Fe is enhanced in the lesion compared with artery wall at levels around 90 ppm. Cu concentrations in the early lesion are a factor of 30 lower, and therefore are unlikely to have the same impact as unregulated Fe in catalyzing free radicals or promoting Cu-medicated LDL oxidation. It has been reported that unregulated Cu is highly pro-oxidative, since it can catalyze free radical formation. Cu can also be anti-oxidative through its role in Cu superoxide dismutase [16]. It has been reported that elevated levels of Fe and Cu were detected in the intima of lesions compared with healthy controls [17]. Stadler et al. [17] have reported that Oxidized lipids and proteins, as well as decreased antioxidant levels, have been detected in human atherosclerotic lesions, with oxidation catalyzed by Fe and Cu postulated to contribute to lesion development. It has been proposed that Zn displaces Fe and Cu from oxidation-vulnerable sites, thereby protect against damage. Furthermore, dietary Zn supplementation in cholesterol-fed rabbits decreases the extent of lesion lipid oxidation and attenuates atherosclerotic burden, despite insignificant changes in lesion Zn. It has also been shown that dietary Cu supplementation significantly decreased aortic atherosclerosis in cholesterol-fed rabbits. The lesions from animals that received the Cu supplement contained fewer smooth muscle cells and fewer apoptotic cells [18]. Our findings are therefore consistent with the hypothesis that in our rabbit model, Cu may play a minor role during the progression of atherosclerosis.

We found in this study that percentage change of Zn was significantly decreased in lung by 8%, kidney by 71% heart by 14% and aorta by 18% compared with control rabbits. These results suggest that Zn may act as an endogenous protective factor against atherosclerosis, perhaps by reducing lesion Fe content, intracellular and extracellular lipids in the intima, connective tissue formation, and smooth muscle proliferation. Furthermore, our results suggest that Zn supplements may completely inhibit the progression of atherogenesis, perhaps by reducing the percentage change of Fe in most of the tissues of HFD rabbits. A study has shown that Zn can reduce the effects of carotid artery injury induced in rats by balloon dilatation, by reducing smooth muscle cell proliferation and intimal thickening [8]. Zn is a co-factor of many enzymes and has been shown to have anti-inflammatory and anti-proliferatory properties. Studies also indicate that Zn is vital to vascular endothelial cell integrity and Zn deficiency causes severe impairment of the endothelial barrier function [9]. Zn is believed to have specific anti-atherogenic properties by inhibiting oxidative stress-responsive transcription factors which are activated during an inflammatory response in atherosclerosis [19]. In other work, test rabbits received a high cholesterol diet with Zn supplements for eight weeks and control rabbits were fed with a high cholesterol diet only for the same period of time. Lesion area analyses showed that the average lesion area was significantly reduced for the rabbits on the Zn-supplement diet [20]. Jenner et al. [7] have reported that Zn has an antiatherogenic effect, possibly due to a reduction in iron-catalyzed free radical reactions. In cholesterol-fed animals, Zn supplementation significantly reduced the accumulation of total cholesterol levels in aorta which was accompanied by a significant reduction in average aortic lesion cross-sectional areas of the animals. Elevated levels of cholesterol oxidation products in aorta of rabbits fed a cholesterol diet were significantly decreased by zinc supplementation. Alissa et al. [21] found that when rabbits were fed dietary supplements of Cu or Zn separately in conjunction with a HFD, aortic atherogenesis was inhibited.

It becomes evident from this study that the changes in trace elements would alter the initiation and progression of atherosclerosis in HFD rabbits. The evidence for the same can be found only in aortic tissue [14], but not in several tissues as in our study. Watt et al. [14] have elucidated the role of trace elements Fe, Zn, Cu and Ca in induced atherosclerosis rabbits. Fe was present in early lesions at concentrations around seven times higher than in normal artery wall. Measurements of localized lesion Fe concentrations were observed to be highly correlated with the depth of the lesion in the artery wall for each individual animal, implying that local elevated Fe concentrations may provide an accelerated process of atherosclerosis in specific regions of the artery. When Fe levels were reduced in the lesion, the progression of the disease was significantly slowed. Zn is depleted in the lesion and is also observed to be anti-correlated with local lesion development. Feeding the rabbits on a HFD with Zn supplements inhibited lesion development, although since no significant increase in lesion Zn levels was measured, this anti-atherosclerotic effect may be indirect. Xi-Ming and Li [22] has reported that published data from 11 countries clearly indicate that the mortality from cardiovascular diseases is correlated with liver iron. It proposes that redox active iron in tissue is the atherogenic portion of total iron stores. Further studies are required to clarify any change in the excretion of trace elements in the stools or urine, and to get the degree of atherosclerosis in HFD rabbits and to correlate the degree of atherosclerosis with the tissue concentration of various trace elements.

## Conclusions

We used AAS to elucidate the role of a HFD on trace elements (Fe, Cu and Zn) in different tissues of HFD rabbits. The findings of this study can be summarized as follows: 1) Percentage change of Fe was significantly increased in lung by 95%, kidney by 7%, heart by 25% and aorta by 33% compared with control rabbits. 2) Percentage change of Cu was significantly decreased in lung by 11%, kidney by 6% heart by 9% and aorta by 16% in HFD rabbits compared with control rabbits. 3) Percentage change of Zn was significantly decreased in lung by 8%, kidney by 71% heart by 14% and aorta by 18% compared with control rabbits.

These results suggest that Fe plays a major role in atherogenesis; it may accelerate the process of atherosclerosis probably through the production of free radicals, deposition and absorption of intracellular and extracellular lipids in the intima, connective tissue formation, smooth muscle proliferation, lower matrix degradation capacity and increased plaque stability. Furthermore, inducing anemia in HFD rabbits may delay or inhibit the progression of atherosclerosis. Cu plays a minor role in atherogenesis and Cu supplements may inhibit the progression of atherogenesis, perhaps by reducing the migration of smooth muscle cells from the media to the intima. Zn plays a major role in atherogenesis and that it may act as an endogenous protective factor against atherosclerosis perhaps by reducing lesion Fe content, intracellular and extracellular lipids in the intima, connective tissue formation, and smooth muscle proliferation. These results suggest that it may be possible to use the measurement of changes in trace elements in different tissues of rabbits as an important risk factor during the progression of atherosclerosis.

## Abbreviations

Fe: Iron; Cu: Copper; Zn: Zinc; HFD (CHO): high fat diet; AAS: atomic absorption spectroscopy; LDL: low density lipoprotein; SOD: superoxide dismutase; NO: nitric oxide; UV: ultraviolet; NOR: normal.

## Competing interests

The authors declare that they have no competing interests.

## Authors' contributions

MAKA, HAA and SAM equally participated in all experiments, analysis and data interpretation and helped to draft the manuscript. The atherosclerotic model used in this study was obtained from the Laboratory Animal Center (College of Pharmacy, King Saud University). The control and HFD was prepared by Research Diet Inc., New Jersey, USA. MAKA and HAA conceived the study and its design, obtained research grants for its development, supervised all technical activities, coordinated data interpretation and wrote the final version of the manuscript. All authors read and approved the final manuscript.
